# (1*R*,2*R*,5*R*,6*R*,9*S*,10*S*,13*S*,14*S*)-1,6,7,8,9,14,15,16,17,17-Decachloro­penta­cyclo­[12.2.1.1^6,9^.0^2,13^.0^5,10^]octa­deca-7,15-diene

**DOI:** 10.1107/S1600536808016231

**Published:** 2008-06-13

**Authors:** Nicole Riddell, Robert McCrindle, Gilles Arsenault, Alan J Lough

**Affiliations:** a Wellington Laboratories, Research Division, Guelph, Ontario, Canada N1G 3M5; b Department of Chemistry, University of Guelph, Ontario, Canada N1G 2W1; c Department of Chemistry, University of Toronto, Ontario, Canada M5S 3H6

## Abstract

The title compound, C_18_H_14_Cl_10_, is a decachlorinated commercial flame retardant. The structure determination confirms the relative stereochemistry. The central eight-membered ring is in a chair-type conformation. In the crystal structure, there are no significant inter­molecular inter­actions and mol­ecules are separated by normal van der Waals distances.

## Related literature

For related literature, see: Garcia *et al.* (1991 ▶); Hoh *et al.* (2006 ▶); Qiu *et al.* (2007 ▶); Sverko *et al.* (2008 ▶); Tomy *et al.* (2007 ▶).
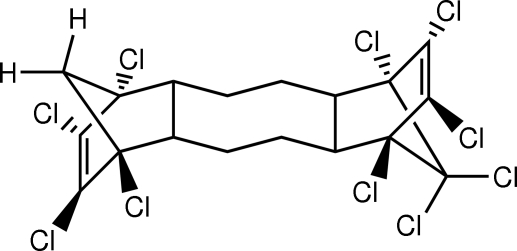


## Experimental

### 

#### Crystal data

C_18_H_14_Cl_10_
*M* *_r_* = 584.79
Orthorhombic, 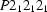

*a* = 11.4341 (2) Å
*b* = 12.9704 (3) Å
*c* = 15.0389 (4) Å
*V* = 2230.34 (9) Å^3^
*Z* = 4
Mo *K*α radiation
μ = 1.25 mm^−1^
*T* = 150 (1) K
0.24 × 0.20 × 0.18 mm

#### Data collection

Bruker–Nonius KappaCCD diffractometer
Absorption correction: multi-scan (*SORTAV*; Blessing, 1995 ▶) *T*_min_ = 0.720, *T*_max_ = 0.804
17031 measured reflections
5087 independent reflections
4585 reflections with *I* > 2σ(*I*)
*R*_int_ = 0.036

#### Refinement

*R*[*F* ^2^ > 2σ(*F* ^2^)] = 0.032
*wR*(*F* ^2^) = 0.072
*S* = 1.04
5087 reflections
253 parameters
H-atom parameters constrained
Δρ_max_ = 0.34 e Å^−3^
Δρ_min_ = −0.32 e Å^−3^
Absolute structure: Flack (1983 ▶), 2207 Friedel pairs
Flack parameter: −0.01 (6)

### 

Data collection: *COLLECT* (Nonius, 2002 ▶); cell refinement: *DENZO*–*SMN* (Otwinowski & Minor, 1997 ▶); data reduction: *DENZO*–*SMN*; program(s) used to solve structure: *SIR92* (Altomare *et al.*, 1994 ▶); program(s) used to refine structure: *SHELXTL* (Sheldrick, 2008 ▶); molecular graphics: *PLATON* (Spek, 2003 ▶); software used to prepare material for publication: *SHELXTL*.

## Supplementary Material

Crystal structure: contains datablocks global, I. DOI: 10.1107/S1600536808016231/bt2716sup1.cif

Structure factors: contains datablocks I. DOI: 10.1107/S1600536808016231/bt2716Isup2.hkl

Additional supplementary materials:  crystallographic information; 3D view; checkCIF report

## Figures and Tables

**Fig. 1.**
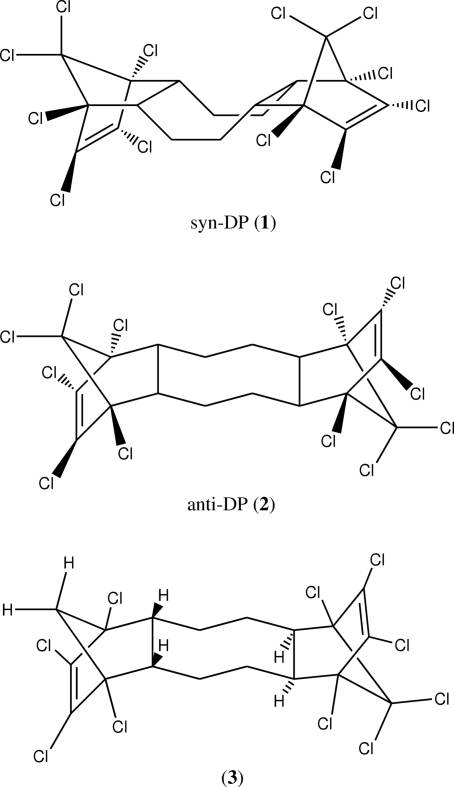
Schematic representation of compounds (1), (2) and (3).

**Fig. 2.**
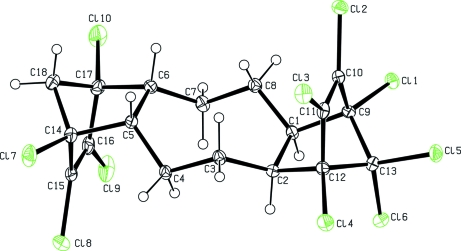
The molecular structure of the title compound. Displacement ellipsoids are at the 30% probability level. H atoms are not shown.

## supplementary crystallographic information

### Comment

Dechlorane Plus (DP) is a commercial chlorinated flame retardant used in styrenic plastics (http://www.inchem.org/documents/ehc/ehc/ehc192.htm) to protect human life and property against fires. The two major components found in the commercial material are known as syn-DP (1R,2R,5S,6S,9R,10R,13S,14S)-[1,6,7,8,9,14,15,16,17,17,18,18- decachloropentacyclo[12.2.1.16,9.02,13.05,10]-octadeca-7,15-diene] and anti-DP (1R,2R,5R,6R,9S,10S,13S,14S)-[1,6,7,8,9,14,15,16,17,17,18,18- decachloropentacyclo[12.2.1.16,9.02,13.05,10]-octadeca-7,15-diene] (see (1) and (2) respectively, Fig. 1). X-ray structure determinations have already been completed on both compounds (Garcia *et al.*, 1991). There is growing evidence that this flame retardant is becoming a significant environmental contaminant (Hoh *et al.*, 2006; Qiu *et al.*, 2007; Tomy *et al.*, 2007). 3–5 Dechlorinated DP species have also been detected in the environment (Sverko *et al.*, 2008) although very little is known about their identity. It is important to identify these compounds if analytical chemists wish to quantify the total presence of DP, including its dechlorinated homologues, in the environment.

We have synthesized the dechlorinated compound (1R,2R,5R,6R,9S,10S,13S,14S)-1,6,7,8,9,14,15,16,17,17-decachloropentacyclo[ 12.2.1.16,9.02,13.05,10]-octadeca-7,15-diene (compound (3); see Fig. 1). GC/MS and ^1^H NMR spectroscopy have confirmed the basic structure of (3) as having the DP-like structure with only 10 chlorine atoms. X-ray structure determination of (3) was required to positively confirm the relative stereochemistry.

### Experimental

The synthesis of compound (3) was carried out at Wellington Laboratories using proprietary methods. The compound was isolated and purified using chromatographic techniques. For single-crystal *X*-ray crystallography, colourless crystals were grown from a solution of (3) in toluene.

### Refinement

All hydrogen atoms were placed in calculated positions with C—H distances of 0.99 and 1.00 Å and they were included in the refinement in a riding-model approximation with *U*_iso_ = 1.2*U*_eq_(C).

### Crystal data

**Table:**

C_18_H_14_Cl_10_	*F*_000_ = 1168
*M**_r_* = 584.79	*D*_x_ = 1.742 Mg m^−^^3^
Orthorhombic, *P*2_1_2_1_2_1_	Mo *K*α radiation λ = 0.71073 Å
Hall symbol: P 2ac 2ab	Cell parameters from 17031 reflections
*a* = 11.4341 (2) Å	θ = 2.7–27.5º
*b* = 12.9704 (3) Å	µ = 1.26 mm^−^^1^
*c* = 15.0389 (4) Å	*T* = 150 (1) K
*V* = 2230.34 (9) Å^3^	Block, colourless
*Z* = 4	0.24 × 0.20 × 0.18 mm

### Data collection

**Table:**

Bruker–Nonius KappaCCD diffractometer	5087 independent reflections
Radiation source: fine-focus sealed tube	4585 reflections with *I* > 2σ(*I*)
Monochromator: graphite	*R*_int_ = 0.036
Detector resolution: 9 pixels mm^-1^	θ_max_ = 27.5º
*T* = 150(2) K	θ_min_ = 2.7º
φ scans and ω scans with κ offsets	*h* = −14→14
Absorption correction: multi-scan(SORTAV; Blessing, 1995)	*k* = −16→16
*T*_min_ = 0.720, *T*_max_ = 0.804	*l* = −19→19
17031 measured reflections	

### Refinement

**Table:**

Refinement on *F*^2^	Hydrogen site location: inferred from neighbouring sites
Least-squares matrix: full	H-atom parameters constrained
*R*[*F*^2^ > 2σ(*F*^2^)] = 0.032	*w* = 1/[σ^2^(*F*_o_^2^) + (0.03*P*)^2^ + 1.117*P*] where *P* = (*F*_o_^2^ + 2*F*_c_^2^)/3
*wR*(*F*^2^) = 0.072	(Δ/σ)_max_ = 0.001
*S* = 1.04	Δρ_max_ = 0.34 e Å^−^^3^
5087 reflections	Δρ_min_ = −0.32 e Å^−^^3^
253 parameters	Extinction correction: none
Primary atom site location: structure-invariant direct methods	Absolute structure: Flack (1983), 2207 Friedel pairs
Secondary atom site location: difference Fourier map	Flack parameter: −0.01 (6)

### Special details

**Table:**

Geometry. All e.s.d.'s (except the e.s.d. in the dihedral angle between two l.s. planes) are estimated using the full covariance matrix. The cell e.s.d.'s are taken into account individually in the estimation of e.s.d.'s in distances, angles and torsion angles; correlations between e.s.d.'s in cell parameters are only used when they are defined by crystal symmetry. An approximate (isotropic) treatment of cell e.s.d.'s is used for estimating e.s.d.'s involving l.s. planes.
Refinement. Refinement of *F*^2^ against ALL reflections. The weighted *R*-factor *wR* and goodness of fit *S* are based on *F*^2^, conventional *R*-factors *R* are based on *F*, with *F* set to zero for negative *F*^2^. The threshold expression of *F*^2^ > σ(*F*^2^) is used only for calculating *R*-factors(gt) *etc*. and is not relevant to the choice of reflections for refinement. *R*-factors based on *F*^2^ are statistically about twice as large as those based on *F*, and *R*- factors based on ALL data will be even larger.

### Fractional atomic coordinates and isotropic or equivalent isotropic displacement parameters (Å^2^)

**Table:**

	*x*	*y*	*z*	*U*_iso_*/*U*_eq_	
Cl1	0.87131 (5)	0.78434 (5)	0.55246 (4)	0.02288 (14)	
Cl2	1.07256 (5)	0.66212 (5)	0.43725 (5)	0.02523 (14)	
Cl3	1.14507 (6)	0.81013 (6)	0.25760 (5)	0.03027 (16)	
Cl4	0.97237 (6)	1.01756 (5)	0.25571 (5)	0.03129 (16)	
Cl5	1.03310 (6)	0.98603 (5)	0.47707 (4)	0.02853 (16)	
Cl6	0.78892 (6)	1.00833 (5)	0.44082 (5)	0.03032 (16)	
Cl7	0.68597 (7)	0.61445 (6)	−0.03570 (5)	0.03760 (18)	
Cl8	0.45590 (8)	0.74067 (6)	0.04705 (6)	0.0468 (2)	
Cl9	0.36780 (6)	0.61715 (7)	0.23695 (6)	0.0441 (2)	
Cl10	0.53873 (7)	0.41158 (6)	0.26610 (5)	0.0406 (2)	
C1	0.7915 (2)	0.78678 (19)	0.37527 (16)	0.0184 (5)	
H1A	0.7177	0.8161	0.4004	0.022*	
C2	0.8244 (2)	0.8505 (2)	0.28950 (17)	0.0195 (5)	
H2A	0.7614	0.9026	0.2790	0.023*	
C3	0.8459 (2)	0.7930 (2)	0.20247 (17)	0.0205 (5)	
H3A	0.8959	0.7325	0.2154	0.025*	
H3B	0.8907	0.8389	0.1624	0.025*	
C4	0.7364 (2)	0.75489 (19)	0.15180 (17)	0.0197 (5)	
H4A	0.6656	0.7817	0.1818	0.024*	
H4B	0.7378	0.7832	0.0907	0.024*	
C5	0.7288 (2)	0.63674 (19)	0.14688 (17)	0.0190 (5)	
H5A	0.8081	0.6103	0.1306	0.023*	
C6	0.6879 (2)	0.57719 (19)	0.23264 (17)	0.0192 (5)	
H6A	0.7506	0.5264	0.2479	0.023*	
C7	0.6627 (2)	0.6394 (2)	0.31572 (17)	0.0220 (6)	
H7A	0.6127	0.5982	0.3560	0.026*	
H7B	0.6188	0.7023	0.2991	0.026*	
C8	0.7757 (2)	0.6710 (2)	0.36566 (17)	0.0205 (5)	
H8A	0.7746	0.6396	0.4257	0.025*	
H8B	0.8440	0.6425	0.3335	0.025*	
C9	0.8936 (2)	0.81438 (18)	0.43944 (18)	0.0186 (5)	
C10	1.0060 (2)	0.77145 (19)	0.40053 (17)	0.0194 (5)	
C11	1.0325 (2)	0.8277 (2)	0.32990 (17)	0.0213 (5)	
C12	0.9372 (2)	0.9089 (2)	0.32037 (17)	0.0212 (5)	
C13	0.9127 (2)	0.93046 (19)	0.42002 (18)	0.0213 (6)	
C14	0.6406 (2)	0.5965 (2)	0.07546 (17)	0.0239 (6)	
C15	0.5203 (2)	0.6380 (2)	0.09832 (19)	0.0258 (6)	
C16	0.4856 (2)	0.5901 (2)	0.17161 (19)	0.0257 (6)	
C17	0.5815 (2)	0.5147 (2)	0.19615 (19)	0.0237 (6)	
C18	0.6255 (3)	0.4837 (2)	0.10393 (18)	0.0252 (6)	
H18A	0.5664	0.4462	0.0683	0.030*	
H18B	0.7000	0.4449	0.1056	0.030*	

### Atomic displacement parameters (Å^2^)

**Table:**

	*U*^11^	*U*^22^	*U*^33^	*U*^12^	*U*^13^	*U*^23^
Cl1	0.0260 (3)	0.0273 (3)	0.0153 (3)	−0.0008 (3)	0.0011 (3)	0.0010 (3)
Cl2	0.0230 (3)	0.0248 (3)	0.0278 (4)	0.0037 (3)	−0.0027 (3)	0.0018 (3)
Cl3	0.0259 (3)	0.0390 (4)	0.0259 (4)	−0.0050 (3)	0.0075 (3)	−0.0007 (3)
Cl4	0.0420 (4)	0.0240 (3)	0.0279 (4)	−0.0119 (3)	−0.0062 (3)	0.0095 (3)
Cl5	0.0351 (4)	0.0252 (3)	0.0253 (3)	−0.0091 (3)	−0.0081 (3)	−0.0011 (3)
Cl6	0.0369 (4)	0.0227 (3)	0.0313 (4)	0.0081 (3)	−0.0059 (3)	−0.0054 (3)
Cl7	0.0549 (5)	0.0391 (4)	0.0189 (3)	−0.0113 (4)	0.0010 (3)	−0.0043 (3)
Cl8	0.0578 (5)	0.0361 (4)	0.0465 (5)	0.0171 (4)	−0.0290 (4)	−0.0059 (4)
Cl9	0.0230 (3)	0.0645 (5)	0.0447 (5)	−0.0044 (4)	0.0035 (3)	−0.0276 (4)
Cl10	0.0526 (5)	0.0323 (4)	0.0367 (4)	−0.0228 (4)	−0.0016 (4)	0.0057 (3)
C1	0.0190 (12)	0.0201 (12)	0.0160 (13)	−0.0019 (10)	−0.0024 (10)	0.0008 (10)
C2	0.0224 (12)	0.0174 (12)	0.0188 (13)	0.0012 (10)	−0.0026 (10)	0.0015 (10)
C3	0.0218 (12)	0.0225 (13)	0.0172 (13)	−0.0028 (11)	−0.0007 (10)	0.0013 (11)
C4	0.0257 (13)	0.0186 (13)	0.0150 (13)	−0.0049 (10)	−0.0029 (10)	0.0019 (10)
C5	0.0192 (12)	0.0185 (12)	0.0193 (13)	0.0009 (10)	−0.0015 (10)	−0.0029 (10)
C6	0.0196 (11)	0.0190 (12)	0.0190 (13)	−0.0015 (10)	−0.0026 (10)	0.0016 (10)
C7	0.0190 (12)	0.0259 (14)	0.0210 (13)	−0.0058 (11)	−0.0003 (10)	0.0021 (11)
C8	0.0223 (13)	0.0207 (13)	0.0186 (13)	−0.0022 (11)	−0.0013 (11)	0.0016 (11)
C9	0.0220 (12)	0.0174 (12)	0.0163 (12)	0.0005 (9)	−0.0007 (10)	0.0028 (10)
C10	0.0191 (12)	0.0195 (12)	0.0197 (13)	−0.0007 (10)	−0.0037 (10)	−0.0010 (10)
C11	0.0195 (12)	0.0233 (13)	0.0210 (14)	−0.0045 (11)	0.0010 (11)	−0.0051 (11)
C12	0.0276 (14)	0.0182 (12)	0.0179 (13)	−0.0045 (11)	−0.0028 (11)	0.0036 (10)
C13	0.0235 (12)	0.0185 (13)	0.0220 (15)	−0.0008 (11)	−0.0020 (10)	−0.0028 (10)
C14	0.0306 (14)	0.0222 (13)	0.0190 (14)	0.0001 (12)	−0.0026 (11)	−0.0034 (11)
C15	0.0246 (13)	0.0214 (13)	0.0315 (16)	0.0026 (11)	−0.0145 (12)	−0.0078 (11)
C16	0.0179 (12)	0.0310 (15)	0.0282 (16)	−0.0047 (12)	−0.0035 (11)	−0.0109 (12)
C17	0.0262 (13)	0.0181 (12)	0.0267 (15)	−0.0055 (11)	0.0001 (11)	−0.0004 (11)
C18	0.0299 (14)	0.0196 (13)	0.0261 (14)	−0.0021 (12)	−0.0017 (12)	−0.0036 (11)

### Geometric parameters (Å, °)

**Table:**

Cl1—C9	1.762 (3)	C5—C14	1.563 (4)
Cl2—C10	1.701 (3)	C5—C6	1.574 (3)
Cl3—C11	1.700 (3)	C5—H5A	1.0000
Cl4—C12	1.758 (3)	C6—C7	1.515 (4)
Cl5—C13	1.775 (3)	C6—C17	1.562 (3)
Cl6—C13	1.766 (3)	C6—H6A	1.0000
Cl7—C14	1.766 (3)	C7—C8	1.550 (3)
Cl8—C15	1.706 (3)	C7—H7A	0.9900
Cl9—C16	1.704 (3)	C7—H7B	0.9900
Cl10—C17	1.771 (3)	C8—H8A	0.9900
C1—C8	1.519 (3)	C8—H8B	0.9900
C1—C9	1.557 (3)	C9—C10	1.518 (3)
C1—C2	1.577 (3)	C9—C13	1.549 (3)
C1—H1A	1.0000	C10—C11	1.324 (4)
C2—C3	1.526 (4)	C11—C12	1.522 (4)
C2—C12	1.567 (4)	C12—C13	1.550 (4)
C2—H2A	1.0000	C14—C15	1.517 (4)
C3—C4	1.547 (3)	C14—C18	1.534 (4)
C3—H3A	0.9900	C15—C16	1.326 (4)
C3—H3B	0.9900	C16—C17	1.514 (4)
C4—C5	1.537 (3)	C17—C18	1.529 (4)
C4—H4A	0.9900	C18—H18A	0.9900
C4—H4B	0.9900	C18—H18B	0.9900
			
C8—C1—C9	112.1 (2)	C10—C9—C13	99.5 (2)
C8—C1—C2	117.9 (2)	C10—C9—C1	108.2 (2)
C9—C1—C2	102.00 (19)	C13—C9—C1	102.2 (2)
C8—C1—H1A	108.1	C10—C9—Cl1	114.42 (17)
C9—C1—H1A	108.1	C13—C9—Cl1	114.65 (18)
C2—C1—H1A	108.1	C1—C9—Cl1	116.00 (18)
C3—C2—C12	110.9 (2)	C11—C10—C9	107.5 (2)
C3—C2—C1	118.9 (2)	C11—C10—Cl2	128.1 (2)
C12—C2—C1	101.96 (19)	C9—C10—Cl2	124.01 (19)
C3—C2—H2A	108.2	C10—C11—C12	107.0 (2)
C12—C2—H2A	108.2	C10—C11—Cl3	127.8 (2)
C1—C2—H2A	108.2	C12—C11—Cl3	125.06 (19)
C2—C3—C4	116.6 (2)	C11—C12—C13	99.4 (2)
C2—C3—H3A	108.1	C11—C12—C2	106.4 (2)
C4—C3—H3A	108.1	C13—C12—C2	103.0 (2)
C2—C3—H3B	108.1	C11—C12—Cl4	116.30 (19)
C4—C3—H3B	108.1	C13—C12—Cl4	115.59 (18)
H3A—C3—H3B	107.3	C2—C12—Cl4	114.32 (18)
C5—C4—C3	112.9 (2)	C9—C13—C12	91.88 (19)
C5—C4—H4A	109.0	C9—C13—Cl6	114.20 (18)
C3—C4—H4A	109.0	C12—C13—Cl6	114.79 (18)
C5—C4—H4B	109.0	C9—C13—Cl5	114.36 (18)
C3—C4—H4B	109.0	C12—C13—Cl5	113.56 (18)
H4A—C4—H4B	107.8	Cl6—C13—Cl5	107.68 (13)
C4—C5—C14	113.8 (2)	C15—C14—C18	100.0 (2)
C4—C5—C6	117.8 (2)	C15—C14—C5	108.1 (2)
C14—C5—C6	101.98 (19)	C18—C14—C5	101.5 (2)
C4—C5—H5A	107.6	C15—C14—Cl7	115.73 (19)
C14—C5—H5A	107.6	C18—C14—Cl7	115.04 (18)
C6—C5—H5A	107.6	C5—C14—Cl7	114.65 (19)
C7—C6—C17	114.7 (2)	C16—C15—C14	107.1 (2)
C7—C6—C5	118.1 (2)	C16—C15—Cl8	127.8 (2)
C17—C6—C5	101.4 (2)	C14—C15—Cl8	124.5 (2)
C7—C6—H6A	107.3	C15—C16—C17	106.8 (2)
C17—C6—H6A	107.3	C15—C16—Cl9	128.3 (2)
C5—C6—H6A	107.3	C17—C16—Cl9	124.4 (2)
C6—C7—C8	112.5 (2)	C16—C17—C18	100.8 (2)
C6—C7—H7A	109.1	C16—C17—C6	108.4 (2)
C8—C7—H7A	109.1	C18—C17—C6	101.5 (2)
C6—C7—H7B	109.1	C16—C17—Cl10	115.6 (2)
C8—C7—H7B	109.1	C18—C17—Cl10	115.51 (19)
H7A—C7—H7B	107.8	C6—C17—Cl10	113.47 (19)
C1—C8—C7	114.0 (2)	C17—C18—C14	92.2 (2)
C1—C8—H8A	108.8	C17—C18—H18A	113.2
C7—C8—H8A	108.8	C14—C18—H18A	113.2
C1—C8—H8B	108.8	C17—C18—H18B	113.2
C7—C8—H8B	108.8	C14—C18—H18B	113.2
H8A—C8—H8B	107.6	H18A—C18—H18B	110.6
